# Faecal microbiota transplantation: a regulatory hurdle?

**DOI:** 10.1186/s12876-017-0687-5

**Published:** 2017-11-28

**Authors:** Frederick Verbeke, Yorick Janssens, Evelien Wynendaele, Bart De Spiegeleer

**Affiliations:** Drug Quality and Registration (DruQuaR) group, Faculty of Pharmaceutical Sciences, Ghent University, Ottergemsesteenweg 460, B-9000 Ghent, Belgium

**Keywords:** Regulatory affairs, Faecal microbiota transplantation, *C. difficile* Infection, Pseudomembranous colitis

## Abstract

During faecal microbiota transplantation, stool from a healthy donor is transplanted to treat a variety of dysbiosis-associated gut diseases. Competent authorities are faced with the challenge to provide adequate regulation. Currently, regulatory harmonization is completely lacking and authorities apply non-existing to most stringent requirements. A regulatory approach for faecal microbiota transplantation could be inserting faecal microbiota transplantation in the gene-, cell- and tissue regulations, including the hospital exemption system in the European Advanced Therapy Medicinal Products regulation, providing a pragmatic and efficacy-risk balanced approach and granting all patients as a matter of principle access to this therapy.

## Faecal microbiota transplantation

The microbiome is no longer considered a passive co-existing community and the idea that these bacteria are idle bystanders clearly belongs to the past. A hallmark is the initiation of the Human Microbiome Project in 2007, aiming to unravel the microbial communities in and on the human body [1]. Exploration of the human microbiome and the relation with the host drastically changed the view towards these microbial cohabitants. Discoveries concerning the role of the microbiome in health and disease provide possibilities towards treatment of several diseases where a profound role is believed to be played by the human microbiome, or a misbalance of the microbiome is at the basis of the condition. In particular, various diseases are linked to an alteration of the gut microbiome, e.g. irritable bowel syndrome, intestinal infections and inflammatory bowel disease [2–7]. Antibiotic-associated nosocomial diarrhoea and colitis are frequently caused by a *Clostridium difficile* infection, a result of gut microbiota dysbiosis [8–10]. The medical signs can range from an asymptomatic carrier state to pseudomembranous colitis and toxic megacolon [11]. Characterised with a mortality up to 40%, influenced by strain and host clinical status, *C. difficile* infection was estimated to be accountable for approximately 500,000 infections and nearly 30,000 deaths in the USA in 2011 [12]. *C. difficile* infection is associated with significant financial consequences as well in the USA [13–17]. Based on the meta-analysis and cost-modelling study by Zhang et al. (2016), the inpatient management alone accounts for an estimated US$ 6.3 billion, a total of 2.4 million days of hospitalization, and the total disease management comes with a cost of US$ 12.4 billion in 2015 in the USA [13]. The occurrence of *C. difficile* infections in Canada are considered to be similar to the infection rates in the USA, with an estimated annual infection rate of approximately 37,900 cases in 2012 and accounting for a financial burden of CAD$ 280 million [18]. In the European Union, 200,000 individuals are estimated to be infected annually, based on the incidence of *C. difficile* in the United Kingdom in 2011–2012 [19]. The most recent cost estimation of *C. difficile* infection in the entire European Union already dates back to 2006, Kuijper et al. estimated the total financial burden caused by *C. difficile* infection to be € 3 billion [20]. From 2006 onwards, several cost estimates have been published, focussing on individual institutions and European individual nations or a limited number of European countries [21–25]. *C. difficile* infections have long been perceived to be a matter of Europe and North-America, whereas the infection rate in Asia remained largely unknown. However, Borren et al. showed, with their systematic review and meta-analysis in 2017, that Asia is affected by a similar infection rate [26]. However, estimates on the financial burden of *C. difficile* infection in Asia are to our knowledge not reported. Additionally, the burden and financial costs liable to *C. difficile* infection in South-America and Africa have not yet been reported in literature. Regarding Oceania, a publication from 2016 estimated the occurrence of *C. difficile* infection in Australia to account for approximately 12,700 cases between 2011 and 2012 [27] with a hospitalisation cost of approximately AU$ 19,000 per patient based on Bond et al. (2017) [28].

**Fig. 1:**
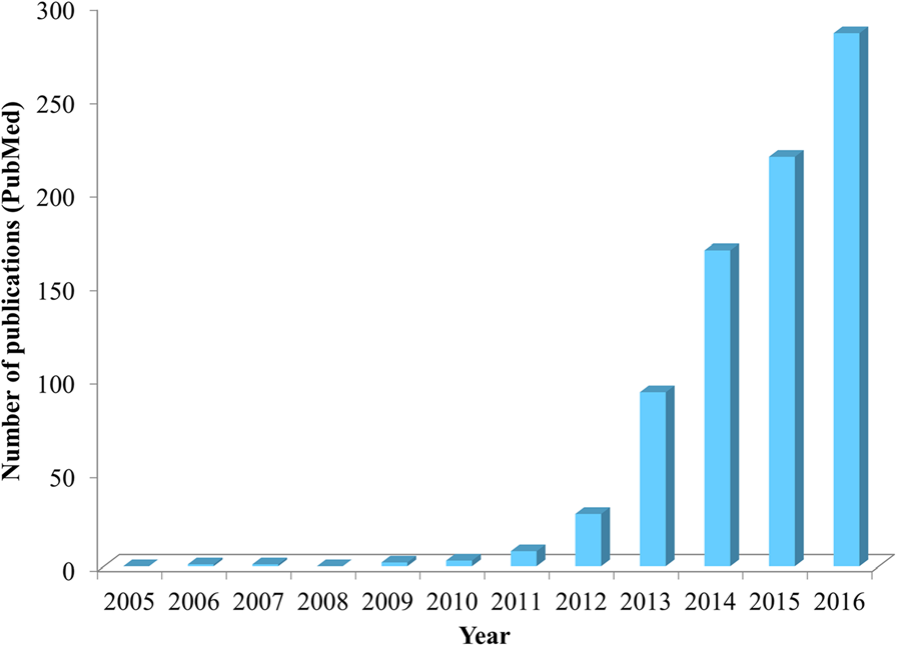
Number of publications per year concerning faecal microbiota transplantation found by a PubMed search (search: faecal microbiota transplantation OR fecal microbiota transplantation)

Initial treatment consists of metronidazole, vancomycin, or fidaxomicin administration. Unfortunately, a considerable number of patients will suffer relapse up to 3 months post-treatment [29–31]. Faecal microbiota transplantation, as a last therapeutic option, consists of the administration of stool from a healthy donor to the patient, hence transferring the donor’s microbiome [32, 33]. The interest of the scientific-medical community for faecal microbiota transplantation, also called faecal bacteriotherapy, or faecal enemas, increased over recent years, as projected in the number of scientific publications (see Fig. 1).

## Evidence for faecal microbiota transplantation

In 2013, the first randomized controlled open-label clinical trial examining the therapeutic advantages of faecal microbiota transplantation versus vancomycin treatment was published by van Nood et al. [34]; they randomly assigned 43 patients (41 patients completed the study) with recurrent *C. difficile* infection to one of the 3 treatment regimens: (I) patients received a short vancomycin treatment (4–5 days of 500 mg 4 times/day), followed by a bowel lavage and faeces transplant via a nasoduodenal tube (17 patients with 1 patient excluded during the study), (II) vancomycin treatment (500 mg 4 times/day during 14 days) (13 patients with 1 patient excluded during the study), and (III) vancomycin treatment (500 mg 4 times/day during 14 days) with bowel lavage at day 4–5 of the treatment (13 patients). Given the distinct effectiveness of faecal microbiota transplantation, compared to the vancomycin treatment, the clinical trial was terminated early, and the planned 40 patients per treatment regimen were not obtained. Patients were considered cured when they remained free of relapse during the 10 weeks following the start of the treatment. In total, 13 patients who received faeces transplantation remained relapse-free during 10 weeks following the start of the treatment, 3 patients experienced relapse and received a second transplant, yielding a total of 15 out of 16 patients that were disease-free at the end of the study. The study group that received vancomycin treatment, without bowel lavage, showed 31% of the patients to remain relapse-free and 23% of the patients remained disease-free in the group that received vancomycin treatment and bowel lavage [34]. Despite these promising results, Vyas et al., warn to be cautious considering the evidence for faecal microbiota transplantation. These authors argue that although ‘*there is ample evidence of the effectiveness of faecal microbiota transplantation from case series and case reports, the lack of sufficient randomized controlled trial data still hinders it from becoming a federally approved or broadly accepted procedure*’. These authors also draw the attention to the study limitations, for example the absence of blinding regarding data collection and the low number of included patients [8]. Additionally, Vyas et al. experience the current screening procedures as potentially detrimental since the screening process takes days to weeks, hence potentially missing intermittently infected (e.g. HIV) donors [8]. For example HIV is known to have a window period, which is the time between the infection and the moment a certain laboratory test will be able to detect the HIV infection. Depending on the applied HIV test, a newly infected faeces donor might be missed or the faeces donor might get infected in the period between HIV testing and faeces donation, hence yielding a serious risk to the patient transplanted with the faeces [35].

After the first randomized controlled trial, as performed by van Nood et al. [34], additional randomized controlled studies have been published [36–40]. A brief discussion of the randomized controlled trials regarding *C. difficile* infection treatment via faecal microbiota transplantation, that followed van Nood et al. [34], is provided. Kelly et al. (2016) compared the effect of autologous faeces transplantation (placebo) versus allogeneic faeces transplantation including a total of 46 patients, who experienced at least 3 bouts of *C. difficile* infection, in the randomized controlled double-blind two-center study. Faeces was transplanted via colonoscopy and Kelly et al. observed that allogeneic faeces transplantation prevented diarrhea up to 8 weeks post-transplantation in 20 out of 22 patients; 15 patients out of 24 were considered cured in the autologous faeces transplant-treatment arm. However, the cure-rate of the patients who received donor stool was liable to the study site where the transplantation occurred; this might be, according to the authors, explained by clinical differences between the 2 study sites [37].

Jiang et al. (2017) examined the effect of fresh, frozen or lyophilized faeces (from healthy donors) when administered via colonoscopy to subjects with at least 3 bouts of *C. difficile* infection in a randomized double-blind study. Jiang et al. concluded that 87% of the patients was *C. difficile* infection-free up to 2 months after the faeces transplantation: 25/25 patients were disease-free when fresh faeces was transplanted, 16/23 were not suffering a *C. difficile* relapse when they received lyophilized faeces and 20/24 patients were considered cured when they received frozen faeces [36]. These observations might seem to be in conflict with the observations of Lee et al. (2016); Lee et al. performed a multi-center study (6 sites) enrolling 232 patients with recurrent *C. difficile* infection in a randomized double-blind non-inferiority trial with 114 patients receiving fresh faeces via enema and 118 patients receiving frozen faeces by enema. In the modified intention-to-treat population, 111 patients received fresh faeces and 108 received frozen faeces and in the per-protocol population, 87 received fresh faeces and 91 frozen faeces respectively. Both the per-protocol population and modified intention-to-treat population demonstrated non-inferiority between fresh and frozen faeces at a non-inferiority margin of 15% regarding diarrhea and adverse events 13 weeks post-treatment [38].

A randomized controlled open-label pilot study was published in 2014 by Youngster et al. where they investigated the impact of the route of administration (via colonoscopy or via a nasogastric tube) in 20 patients with recurrent *C. difficile* infection, equally divided over the 2 treatments. Youngster et al. concluded that the route of administration appears to yield comparable diarrhea resolution without relapses up to 8 weeks post-transplantation and improved self-reported health scores with an overall cure rate of 90% [39].

Cammarota et al. (2015) performed an open-label randomized controlled trial to compare the effect of faeces transplantation via colonoscopy (consisting of vancomycin treatment 125 mg 4 times/day, bowel lavage at day 1–2 and subsequent faeces transplantation) versus vancomycin treatment (10 days of 125 mg 4 times/day and a subsequent pulse-treatment with 125–500 mg/day during at least 3 weeks every 2–3 days) in a patient population (39 patients were randomized over the treatments) with recurring *C. difficile* infection, hence more or less resembling the study of van Nood et al. [34]. Patients were considered cured by Cammarota et al. when they remained relapse-free 10 weeks after the treatment. Comparable with van Nood et al. [34], the study by Cammarota et al. was terminated after an interim analysis with finally 90% (18/20 patients) of the patients that received (one or more) faeces transplants considered cured and 26% (5/19 patients) of the vancomycin treatment group considered cured [40].

The single-center, open-label, randomized trial performed by Hota et al. (2017) investigated the effectiveness of vancomycin treatment versus faecal microbiota transplantation for recurrent *C. difficile* infection (at least 2 laboratory confirmed episodes of *C. difficile* infection and prior vancomycin treatment during these bouts). The number of participants was limited following an interim analysis, yielding 16 patients allocated to the faecal microbiota treatment arm (faeces transplantation via an enema preceded by a 14 days vancomycin treatment consisting of 125 mg every 6 h) and 12 patients received a gradually decreasing, vancomycin treatment during 6 weeks (125 mg vancomycin every 6 h the first 2 weeks and gradual treatment tapering the consecutive 4 weeks). Patients were considered cured if no *C. difficile* relapse occurred 120 days following the intervention. Finally, 56% (9/16) of the patients that received faecal microbiota transplantation and 42% (5/12) of the patients under vancomycin treatment experienced a *C. difficile* relapse, making the authors to conclude that a single-faecal microbiota transplantation is not significantly different from vancomycin treatment when patients with recurrent *C. difficile* infection are confronted with a new *C. difficile* episode [41].

Not only randomized controlled trials, but also various systematic reviews and meta-analyses covering faecal microbiota transplantation for (recurrent) *C. difficile* infection have also been published [42–50] with the most recent one published in 2016 [42].

Gough et al. (2011) evaluated the effect of faecal microbiota transplantation on recurrent *C. difficile* infection. To do so, they included in their systematic review 317 patients, originating from 8 countries over a period of 53 years, from a total of 27 case series and case reports. They concluded that (I) 92% of all included patients experienced disease resolution, (II) faecal microbiota transplantation is therefore a suitable treatment for recurrent *C. difficile* infection, (III) treatment effectiveness is highly depend on for example the route of administration and the pre-treatment the patient receives prior to faeces transplantation, and (IV) identified several limitations, for example: the lack of uniformity across the studies (for example definitions), the lack of standardisation (for example pre-transplantation treatment), heterogeneity across patients, potential publication bias and the need of randomized controlled trials [47].

Comparable, Sofi et al. only included retrospective studies and case reports in their systematic review from 2012 to investigate the effect of faecal microbiota transplantation for *C. difficile* infection. However, no major overlap is apparent between the included publications by Sofi et al. [49] and Gough et al. [47]. From a total of 25 publications, 289 patients were included by Sofi et al. for data analysis, demonstrating a total disease resolution of approximately 91%. The authors mention as limitations of their meta-analysis: possible publication bias, lacking uniformity in the faecal microbiota transplantation procedure (for example different quantities of infused faeces) and missing variables (for example time between diseases onset and treatment). Nevertheless, they conclude that faecal microbiota transplantation is an effective and safe treatment for *C. difficile* infection [49].

In the systematic review performed by Guo et al. (2012), the authors also aimed to investigate the evidence for faecal microbiota transplantation as treatment for *C. difficile* infection. As the included publications majorly converge with the publications applied by Sofi et al. [49] and Gough et al. [47], this meta-analysis is also solely based on case series and case reports. In total, 7 publications are retained by the authors, yielding a total of 124 patients. As previous meta-analyses concluded, Guo et al. also conclude that faecal microbiota appears to be a promising treatment for *C. difficile* infection since approximately 80% of the included patients experienced disease resolution. The limitations they identify are in line with previous authors, encompassing among others, the need for randomized controlled trials [50].

The systematic review and meta-analysis of Kassam et al. in 2013 also aimed to investigate the evidence for faecal microbiota transplantation as treatment for *C. difficile* infected individuals. The meta-analysis was conducted on the data from 273 patients, extracted from 11 retained publications, which are all included in the study of Sofi et al. [49] and show a large convergence with Gough et al. [47] and Guo et al. [50]. Kassam et al. provide in their publication the argumentation why the meta-analyses of Gough et al. [47] and Guo et al. [50] have, to their opinion, limitations. The authors also demonstrate a cure rate above 90% and do also conclude that faecal microbiota transplantation is a promising treatment for *C. difficile* infection and do also address the need for randomized controlled trials [48].

O’Horo et al. (2014) extended the scope of their systematic review and meta-analysis towards 8 different treatments (metronidazole, vancomycin, fidaxomicin, nitazoxanide, rifampin, immunoglobulins, probiotics and faecal microbiota transplantation) for *C. difficile* infection. Considering faecal microbiota transplantation, a total of 33 publications with the majority of these publications being included in the previously discussed systematic reviews, are discussed. The data of 609 patients were analysed in this meta-analysis; data obtained predominately from case reports or case series and only 2 prospective trials and 1 randomized controlled trial [34]. In conclusion, O’Horo et al. find faecal microbiota transplantation a feasible option for *C. difficile* treatment, but they pinpoint the need of more randomized controlled trials [45].

Several other authors also performed meta-analyses following previous meta-analyses and continued to include new studies in their systematic reviews. For example Cammarota et al. also performed a systematic review in 2014, including 20 case series, 15 case reports and 1 randomized controlled trial, i.e. published by van Nood et al. [34], which have majorly already been included in previous systematic reviews. The authors performed their meta-analysis on 536 patients in total and concluded that 87% of them benefited from faecal microbiota transplantation for *C. difficile* treatment [44]. Dodin et al. identified at their turn in 2014, 20 clinical reviews, 6 case reports, 2 clinical trials, 1 randomized controlled trial and 1 meta-analysis in their systematic review. They also conclude that faecal microbiota transplantation has a place in the treatment of *C. difficile* infection, but several unmet needs needed to be addressed to obtain additional evidence, especially the need of additional clinical trials/randomized controlled trials [46].

Bagdasarian et al. investigated both the preferred method of diagnosis and treatment of *C. difficile* infection in their 2015 systematic review. However, no meta-analysis was performed and they base their conclusions on the publications they retrieved. Regarding *C. difficile* treatment, they conclude that depending on the severity of the infection, metronidazole, vancomycin or fidaxomicin are preferred. According to the authors, faecal microbiota transplantation is supported by increasing evidence [43].

The most recent meta-analysis dates back to 2016 and was performed by Li et al. The authors focussed in their publication on the long-term effects and safety of faecal microbiota transplantation for *C. difficile* treatment. They included 611 patients from in total 18 publications, several already included in aforementioned meta-analyses. Among the included studies, no randomized controlled trial was included and the included studies are retrospective, prospective case series or retrospective cohort studies. Finally, they argue that faecal microbiota transplantation has a primary cure rate of approximately 91% with an overall recurrence rate of approximately 6%. Serious adverse events were not identified by Li et al., with the most frequent adverse events being classified as transiently and self-limiting. As other authors, they also advocate the need for randomized controlled trials to increase the evidence [42].

In summary, these systematic reviews and meta-analyses conclude that, despite some publication-specific shortcomings, faecal microbiota transplantation for (recurrent) *C. difficile* infection is a promising treatment. However, some systematic reviews and meta-analyses also point towards the need of more high-quality studies, for example cohort studies and randomized controlled trials to confirm long-term effects (both efficacy and safety).

Other conditions, for example inflammatory bowel disease and more specific ulcerative colitis, have also been subjected to systematic reviews and meta-analyses [51–55]. In general, the various authors conclude that faecal microbiota transplantation might be an effective treatment of ulcerative colitis, but additional well-designed studies, for example randomized controlled trials, are essential to obtain more evidence.

The increasing evidence has urged several expert groups to issue guidelines on the indications for faecal microbiota transplantation and standardized methods to perform the transplantation [56–60].

## Faecal microbiota transplantation: Viewpoint regulatory authorities

In 2017, a globalized, common position regarding faecal microbiota transplantation regulations is still lacking. The entire spectrum of regulatory statuses is currently present, ranging from non-existing to strictly regulated. In 2012, the Competent Authorities on Substances of Human Origin Expert Group of the European Union concluded that faeces transplantation is not covered by the European Human Tissue Directive 2004/23/EC [61, 62]. The discussion was reopened in June 2014 and the regulatory uncertainty in the European Union was readdressed by the Competent Authorities on Substances of Human Origin Expert Group during discussions held concerning the regulatory status of human milk and faeces donation [63]. Consecutively, the European Commission vowed in consulting their legal service asking (I) ‘*Should human milk and/or faeces be considered as falling within the scope of the Human Tissue Directive 2004/23/EC’* and (II) ‘*If not, could such substances be regulated under the mandate laid down in Article 168(4) of the Treaty on the Functioning of the European Union?*’ [61]. To comply with Directive 2004/23/EC, the substance must be a ‘Tissue’ or ‘Cell’ and be intended for ‘Human application’. According to the European Commission, both criteria can be considered as being met since both human milk and faeces contain ‘Cells’. Because faeces and human milk are not administered because of the presence of human ‘Cells’; they are administered for the other components (for example nutrients in the case of human milk or bacteria/bacterial components in the case of faeces), the European Commission concluded that faeces transplantation is not covered by Directive 2004/23/EC [61]. Consecutively, the European Commission argues that individual Member States are free to regulate faecal microbiota transplantation on a national level [61, 64], however only few EU member states have imposed faecal microbiota transplantation regulatory demands until now and most European countries have no regulation on faecal microbiota transplantation at all. For example, the Superior Health Council of Belgium argued in 2015 that currently unknown active substance(s) urges them to consider faecal microbiota as a human body tissue [65].

However, the Superior Health Council of Belgium also acknowledged in March 2015 that, in the future, faecal microbiota can evolve towards the status of medicine when the product becomes a more specified product concerning the composition of the active substance(s) or the possibility of an industrial production process [65]. Another position is reflected by the National Security Drug Agency of France (Agence National pour la Sécurité du Médicament), which issued in March 2014 that faecal microbiota is to be considered as a pharmaceutical drug [66, 67].

Austria has the most liberal point of view: the Federal Office for Safety in Health Care argues that faecal microbiota transplantation is to be considered as a therapeutic intervention, explicitly not applying to the definition of a drug as specified in the Austrian Medicines Act, neither being subject to the Medical Devices Act or to the Austrian Transplantation Act [68]. To provide medical practitioners some guidance, the Austrian Society of Gastroenterology and Hepatology, in cooperation with the Austrian Society of Infectious Disease and Tropical Medicine and the Austrian Agency for Health and Food Safety, issued in 2014 a faecal microbiota transplantation consensus guideline. Given the lack of sufficient evidence for other conditions than *C. difficile* infection, the consensus guideline recommends the use of faecal microbiota transplantation for other indications under the setting of clinical trials [68, 69]. Hence, faecal microbiota transplantation is currently available in Austria for patients suffering from *C. difficile* infection, while other indications can be treated under the settings of a clinical trial.

The United States of America Food and Drug Administration stated in May 2013 that faecal microbiota transplantation is considered to meet the description of a drug, hence requiring an Investigational New Drug application prior to conducting a faecal microbiota transplantation. Various stakeholders addressed their concerns, indicating this time-consuming action might restrain patient’s access to this treatment. The Food and Drug Administration (USA) acknowledged this concern 6 weeks later and until today, the necessity to file an Investigational New Drug application was revoked for recurrent *C. difficile* infections. Although faecal microbiota transplantation retains the drug status, currently this policy of tolerance remains [70]. In summary, patients suffering from *C. difficile* can be treated by faecal microbiota transplantation in the USA, but the situation can be considered precarious as the Food and Drug Administration can revoke their policy of tolerance.

## Finding the right balance

Given the interaction of the gut microbiota with various physiological processes, both in health and disease, some argue that the gut microbiota should be envisaged as a separate, individual organ. Hence, faecal microbiota transplantation can be considered in that point of view as an organ transplantation with corresponding screening standards [8, 71, 72].

Smith et al. strikingly compared in 2014 current faecal microbiota transplantation risks with the HIV and hepatitis infections originating from blood-donation during the 1970 and 1980’s, a period HIV was largely unknown [70]. Considering the possible effect of the microbiome in a variety of diseases, this concern might in future years be proven significant [48]. Hence, a careful and potentially ‘over-regulating’ approach is justified as a safety precaution regarding unknown possible long-term adverse effects. However, given the acute character and the undeniable mortality rates, the apparent therapeutic superiority of faecal microbiota transplantation compared to standard antibiotic therapy and the persistent suffering of the patient affected by *C. difficile*, hindering patients access to the faecal microbiota transplantation treatment can be considered as inhumane.

Alternative legislation has been proposed by Sachs et al. in 2015. They propose to construct a hybrid regulatory approach, comparable as performed for blood and cord blood [73]. Smith et al. consider the current faecal microbiota transplantation regulation as both over- and under-regulating as *C. difficile* infections can be treated by faecal microbiota transplantation without stringent limitations. In the USA, the Investigational New Drug applications necessary for other disorders imposes a distinct barrier for clinical researchers to examine the usefulness of faecal microbiota transplantation for other pathologies [70]. Regulators should keep in mind that, contrary to other treatments, faecal microbiota transplantation can be performed by the patient itself without much obstacles. The procedure can easily be conducted using standard kitchen devices and already do-it-yourself faecal microbiota transplantation videos are freely available [74]. The author of one of the videos also explicitly mentions that he was suffering for over 12 years from ulcerative colitis and provides viewers with a do-it-yourself faecal microbiota transplantation manual; this video was aired in 2013 and attracted around 80,000 views 4 years later [61]. In brief, the video describes and demonstrates how faeces from a donor is obtained, blended in a kitchen blender (the author luckily mentioned to use a dedicated blender) with deionised water with salt added to obtain home-made saline, how the resulting suspension is to be sucked into a rectal syringe and how the faeces suspension must be retained in the gut to allow it to reach as much of the colon as possible. However, do-it-yourself faecal microbiota transplantation imposes various risks, for example: health-risks, legal liability and possible long-term effects. In the Youtube® video, the author mentions that relatives are the most appropriate donors since ‘*you can trust that you know their medical history, hopefully you can at least*’, nevertheless the author of the Youtube® video advices to perform several laboratory tests to assess the donor’s health status [74]. Although, it can be expected that some patients do not wish to test their faeces donor because ‘they trust their donor is healthy’, hence exposing themselves to possible infections the donor might not be aware of because he/she was infected lately with for example HIV or Hepatitis, among others. Additionally, donor assessment should be strictly reserved to a person with a medical training.

The video also advices on the use of certain pharmaceutical drugs, near the end of the video, to accommodate with some inconveniences faecal microbiota transplantation might cause when administered via a rectal syringe, for example prednisone in advance of the faecal microbiota transplantation. Altering/starting medication schemes can impose various health risks and must be conducted by medically trained professionals.

The lack of anonymity of faeces donation, and the largely unknown long-term effects various faecal microbiota transplant experts put forward, can have (legal) liability issues in the future which do-it-yourself patients are most probably not aware of.

Even more striking is a video from 2013 where a patient testifies how she received as a 10 year old girl, do-it-yourself faecal microbiota transplantation by her mother [75].

Hence, denying patients access due to a too stringent regulation can redirect desperate patients towards these uncontrolled and potentially harmful do-it-yourself procedures, or to quote the author of the do-it-yourself faecal transplant book ‘Poop Power – story and guide book’: ‘*While I would have preferred to work with a doctor, and almost did, the process is cumbersome and many doctors do not want to do this procedure because of obstacles put in place by hospitals and government regulations. In the meantime this procedure can be done at home with proper guidance*’ [76].

Another consideration that should be reflected in the regulation of faecal microbiota transplantation are the possible advancements probably made in near future [8]. Administering selected microbiota as a capsule differentiates it from a medical intervention, meeting more the definition of a pharmaceutical drug. Evolving away from the current faecal material, obtained from donors, towards synthetic/purified faecal microbiota transplantation material can lower the donor-associated risks, e.g. pathogens, and pave the way for industrial production [77]. This potential future for faecal microbiota transplantation strengthens the urgent need of an applicable and suitable regulatory status.

## A pragmatic regulatory approach for a quickly evolving therapy

The current situation, as well the potential evolutions in the nascent field of faecal microbiota transplantation, possibly allowing a shift from faecal transplantation towards more defined products containing microorganisms, urgently calls for a regulatory frame which grants patients access to faecal microbiota transplantation as a matter of principle. An urgent need for a regulatory framework is persistent for reasons of clarity, given the vast amount of poor quality studies and case reports apparent until now in literature and the lack of a standardized procedure. Baxter et al. strikingly draw the attention towards the absence of a structured follow-up of faecal microbiota transplantation patients [78].

Structured donor selection, quality control and follow-up of faecal microbiota transplantation could be based on blood and blood products (e.g. cord blood) regulations as previously argued by Sachs et al. [73]. Despite various resemblances between blood (products) and faeces, for example the need of donors and the risk of infecting the recipient, we identify several properties of faeces that urge another regulatory approach.

The exact mechanism of action of faecal microbiota transplantation is still to be resolved. In other words, the exact contributing micro-organisms and/or their metabolites are still to be discovered. The functional components of blood (products) on the other hand, are more straight-forward and well-known, for example: thrombocytes are isolated and administered to a patient with thrombocytopenia.

Until now blood cannot be produced in vitro and donor involvement is mandatory. Faecal microbiota transplantation has the opportunity to evolve towards a defined bacteria-based product, a ‘bacteriaceutical’, hence faecal microbiota transplantation can be considered as the initial step. In a certain way, faecal microbiota transplantation can be compared to opium. Before the isolation and chemical synthesis of opioids, patients received the opioids as a crude/mixture/extract from *Papaver somniferum*. Comparably, the necessary gut bacteria/bacterial components for patients suffering intestinal dysbiosis are currently administered as being part of faeces, but these necessary microbiota and/or their products might in the near future be identified and administered without faeces being involved.

The general disease-healing potential of faecal microbiota transplantation is just being explored with currently ulcerative colitis as the most promising example.

Hence, the regulatory framework must accommodate evolutions of the product and must encourage conducting clinical trials to investigate the possible advantages of faecal microbiota transplantation for other conditions.

Therefore, an interesting possibility to regulate faecal microbiota transplantation, given its distinct complexity, could be based on current regulations. Extending the scope of the gene-, cell-, and tissue therapeutics allows inclusion of faecal microbiota transplantation in this global product group, e.g. the Advanced Therapy Medicinal Products in the European Union, this regulatory approach will allow to meet the necessities that are defined in Table 1 and will facilitate possible, future advancements. As a matter of clarity, the argumentation why faecal microbiota transplantation must be regulated based on the cell-, tissue-, and gene-regulation has been limited to the situation in the European Union. However, a comparable argumentation can also be constructed based on the legal situation of cell-, tissue-, and gene-therapeutics in other countries, e.g. USA, Japan, Canada or Australia.

**Table 1:** Principles to be considered for faecal microbiota transplantation regulation

Principle	Explanation
Regulatory harmonization	The regulation of faecal microbiota transplantation must be comparable around the world. Some countries have regulated faecal microbiota transplantation ad interim (e.g. by regulatory discretion in the USA), some countries have a liberal faecal microbiota transplantation regulation (e.g. Austria) and various countries do not have any legal framework at all regarding faecal microbiota transplantation (e.g. various European Union member states). Hence, safe and regulated access to faecal microbiota transplantation currently still majorly depends on the country where the patients are living in.
Patient Empowerment	Patients should be empowered and allowed to make informed decisions. Providing patients with adequate, scientifically sound information to allow them to, together with their physician, weigh the possible benefits and risks attached to faecal microbiota transplantation and to take an informed decision. From the point of view that every person must be allowed self-determination regarding their health.
Quality	Faecal microbiota transplantation must be conducted with faeces that meets rigorous quality standards (e.g. absence of pathogens and infectious transmittable diseases) to limit the risks to the recipient.
Donor anonymity	Frequently, relatives/partners are donors given their comparable microbiome. Since long-term adverse events seem until today unclear, one could question donors to be blamed when such long-term adverse events would occur. Hence faeces donation must be conducted in an anonymous, but traceable, way.
Efficacy	The efficacy of faecal microbiota transplantation must be monitored by an independent organisation (e.g. the competent authorities) to safe-guard patients and to allow the practise of evidence-based medicine.
Information	All stake holders must be provided with adequate information. For example, the patient must obtain all relevant information available and must be made aware of possible long-term risks, possibly currently unknown.
Pharmacovigilance	Adequate monitoring of patients though time must be maintained to detect e.g. late adverse effects and to allow pharmaco-epidemiology and pharmaco-economics.

The European Advanced Therapy Medicinal Products Regulation came into act in 2007 and was issued to respond to the fast scientific evolutions in the field of tissue engineering, gene therapy and somatic cell therapy, giving the centralized European Medicines Agency the authority to regulate these products in the entire European Union. Compared to other pharmaceutical drugs, these cell-, tissue- and gene-based products have more specific needs regarding assessment of efficacy, safety and quality.

Currently, the European Advanced Therapy Medicinal Products regulation can regulate various products: gene therapy medicinal products are considered products aiming to alter/repair/replace gene(s) or the effect is linked to the administered nucleic acid(s) or the product resulting from the genetic expression [79], whereas tissue- and cell-based therapy medicinal products in essence contain human or animal cells or tissues, viable or non-viable, engineered or not, with or without additional products (e.g. bio-molecules), with the aim to exert a pharmacological, immunological or metabolic effect [78]. On a legal basis, the gene-, cell-, and tissue-regulation appears to be able to encompass the complexity of faecal microbiota transplantation, since faeces is obtained from a human donor and is transferred to a human receptor, hence encompassing an infection risk. The human microbiome, more specific the gut microbiome, is considered as a necessary part of our body since dysbiosis of the gut microbiome is linked to a variety of diseases, as for example *C. difficile* infection. Currently, this microbiome-component is administered as the complex mixture faeces is. A historical parallel can be identified with the evolution of recombinant proteins, with insulin serving as an example. Insulin was discovered in the early twentieth century as the component that regulates the blood glycaemia, with a Nobel prize awarded in 1923 for its discovery, but it would take until the 1980’s [80] before the first synthetic, human insulin was available to patients. In the intermittent decades, diabetics were administered insulin from bovine and/or porcine origin [81]. Comparably, the necessary components of the faecal transplant, although from microbial origin and thus currently not directly falling under the gene-, cell-, and tissue regulation, might soon be identified and administered as such, without the need of transplanting complete faeces. The gene-, cell-, and tissue regulations were established to cope with the potential fast evolutions of these therapeutics. Analogue, this regulation is able to cover the possible advances made in faecal microbiota transplantation, since this regulation has been written with an open view to remove as much as possible legal obstacles for the fast bench-to-bed transition of promising treatments.

Additionally, a majority of the risk/benefit balance concerning faecal microbiota transplantation, e.g. traceability, quality, including Good Manufacturing Practice, pharmacovigilance and information to the patients, are already covered by the gene-, cell- and tissue regulation of the European Union [82]. For example, the regulation defines the conditions that must be met when human cells and tissues are donated: both the donor and recipient must be anonymous, altruism of the donor must be the driving force to donate, hence donation must be voluntary and unpaid. At the same time, a key concern of current faecal microbiota transplantation, i.e. the long-term effects, is faced by the legal demand to establish appropriate measures to monitor any possible adverse reaction over time. Hence, a suitable risk management system capable of addressing risk identification, characterisation, prevention and minimisation plus being capable of assessing the therapy effectiveness must be in place. Currently, if faecal microbiota transplantation is performed, it remains unclear how traceable the faeces transplantation has been performed since no clear legal demands are defined. The Advanced Therapy Medicinal Product regulation defines strict traceability demands that need to be met: patient, donor, the product/starting material must be recorded in a traceable manner for at least 30 years after the treatment has been performed. The facility where the product has been administered must be able, via traceability, to link the patient to the specific product administered. According to the current regulation, if the holder of the marketing authorisation would be unable to fulfil this legal demand (e.g. bankruptcy), the data must be transferred to the European Medicines Agency.

Besides, this legal framework provides in a formal, tailored clinical trial status, possibly leading to a formal marketing authorization, but also in the possibility to flexibly exercise the hospital exemption [82], under strictly defined conditions. The hospital exemption in the European Union is applicable when an Advanced Therapy Medicinal Product is prepared on a non-routine basis, but with specific quality standards, in a hospital setting for administration in the same European Union state. The aim of this hospital exemption is to meet a medical need of a specific patient and is administered to this patient under the responsibility of the medical practitioner [82].

Other stake-holders, for example medical practitioners, would also benefit by regulating faecal microbiota transplantation according to such a legal framework. The most straight-forward advantage would be liability when faecal microbiota transplantation is regulated by law. Currently, several countries do not have such laws, hence potentially exposing medical practitioners to law suits due to the lacking legal framework. In first instance, expanding the scope of such a legal framework towards faecal microbiota will, because of the hospital exemption, not cause an immediate cessation of faecal microbiota transplants [67]. Moreover, such a legal framework would encourage the initiation of clinical studies, e.g. to provide evidence on the treatment of ulcerative colitis via faecal microbiota transplantation. On a European level, marketing authorisation of faecal microbiota for a given disease would immediately grant all citizens of the European Union access to the treatment [83], avoiding unnecessary replication of clinical trials due to different regulatory demands per country. Therefore, we strongly advocate competent authorities around the world to cooperate on the regulatory framework of faecal microbiota transplantation, since already various countries have adopted, or are working on, cell-, tissue-, and gene-therapeutic regulations, for example India [84], Australia [85], Canada [86], USA [87] and Japan [88].

## Conclusion

Including faecal microbiota into the gene-, cell-, and tissue class will meet the demands of patients, health care practitioners, competent authorities and industry. Faecal microbiota transplantation challenges the current regulatory frameworks due to its complex nature. Therefore we propose to expand the scope of the various cell-, gene-, and tissue regulations towards faecal microbiota. Applying such a regulatory approach globally, will give patients access to this potentially life-changing and -saving therapy.
